# Recycling of Periwinkle Shell Waste as Partial Substitute for Sand and Stone Dust in Lightweight Hollow Sandcrete Blocks towards Environmental Sustainability

**DOI:** 10.3390/ma16051853

**Published:** 2023-02-24

**Authors:** Oluwarotimi M. Olofinnade, Joshua U. Anwulidiunor, Kunle E. Ogundipe, David A. Ajimalofin

**Affiliations:** 1 Department of Civil Engineering, College of Engineering, Covenant University, Ota 112233, Nigeria; 2 cidb Centre of Excellence & Sustainable Human Settlement and Construction, Department of Construction Management & Quantity Surveying, Faculty of Engineering and the Built Environment, University of Johannesburg, Johannesburg 2092, South Africa

**Keywords:** periwinkle shell, hollow sandcrete block, compressive strength, water absorption, sustainable construction, recycling, waste management

## Abstract

Global consumption of nonrenewable natural aggregate for construction activities is now becoming a significant concern. Reusing agricultural or marine-based wastes could offer a promising alternative to achieve natural aggregate conservation and a pollution-free environment. This study investigated the suitability of using crushed periwinkle shell (CPWS) as a reliable constituent material for sand and stone dust in producing hollow sandcrete blocks. The CPWS was used to partially substitute river sand and stone dust at 5, 10, 15 and 20% in sandcrete block mixes using a constant water–cement ratio (w/c) of 0.35. The weight, density and compressive strength of the hardened hollow sandcrete samples were determined after 28 days of curing along with the water absorption rate. Results showed an increase in the water absorbing rate of the sandcrete blocks as CPWS content increased. Mixes containing 5% and 10% CPWS substitute for sand with 100% stone dust surpassed the minimum targeted strength of 2.5 N/mm^2^. The compressive strength results suggested that CPWS is most suitable to be deployed as a partial substitute for sand as a constant stone dust material, thus imply that the construction industry can achieve sustainable construction with agro or marine-based wastes in hollow sandcrete production.

## 1. Introduction

Masonry work is an important part of building construction and is a process of connecting building units together with binding agents to achieve the building. For decades now, one of the many conventional materials used for masonry works and construction has been fabricated composite blocks or bricks [1]. Blocks are usually precast materials used in the construction of both residential and public building structures in many countries of the world. The common types of blocks used for building construction have varied material compositions [2,3]. Cement, fine and coarse aggregates and water are the major components in block work production [2]. For instance, there are composite blocks made with only cement and sand used as fine aggregates, referred to as sandcrete blocks, while there are those made with cement, sand and the addition of coarse aggregate. This type of composite block is referred to as a concrete block [3,4]. The concrete blocks are used in major civil engineering construction work, and can be moulded in situ or precast to form different shapes [5]. Further, according to Oyekan and Kamiyo [6], in most developing nations of the world, especially in Africa, sandcrete blocks are the commonly used material for building construction, especially for residential purposes. Hollow sandcrete blocks are a type of conventional sandcrete block used in mostly residential and commercial buildings. A huge volume of nonrenewable resources, including cement, is used in the production of sandcrete blocks, thus there is a need to find an alternative means of reducing these material contents to achieve raw resources conservation and reduce carbon emissions [7,8].

Similarly, there has been an increasing demand for natural resources for infrastructure due to the increasing need for housing and infrastructure caused by urbanization, population growth and economic development [7,9,10]. Sandcrete blocks are one of the building materials required in high volume for housing construction [6]. However, studies have shown that continuous exploitation of river sand as material for production of composite building materials such as sandcrete blocks, mortar and concrete contributes significantly to natural resource depletion, causing erosion of coastal lands as well as flooding [11]. To lessen these negative impacts, as Kumar et al. [12] opined, researchers are continually investigating means of utilizing alternative materials as partial or whole substitutes for the cement, fine aggregates and coarse aggregates used in the production of these composite materials. This would enable the construction industry to achieve sustainable ecofriendly construction, resource conservation as well as low-cost design and responsible consumption [8,12]. Research studies have investigated several waste materials as viable alternatives to natural aggregates in production of building materials such as sandcrete blocks. These waste materials include construction rubble wastes, discarded glass, plastic wastes, waste bricks, tyres, and agricultural waste materials such as palm kernel shell [13,14,15,16,17]. Reports from these studies indicated the possibility of including these materials as constituents in blocks/bricks without compromising the quality. Furthermore, some studies have shown that utilisation of waste materials having pozzolanic composition, such as sawdust, fly ash, sewage sludge ash, rice husk, metakaolin, waste glass, ceramic and tile wastes and waste clay brick, can be used as substitutes for cement to make good-quality blocks and bricks for construction [14,18,19,20,21,22].

Meanwhile, beyond helping to achieve a sustainable clean environment through recycling of these waste materials, studies are also looking at ways of providing affordable or low-cost building materials for housing without compromising their quality [8,23]. As earlier mentioned, sandcrete blocks are widely used in the construction of housing for residential purposes [24,25]. As noted in a recent study by Ogundipe et al. [23], one of the present problems in the construction industry is affordability in housing, especially in developing countries, as previously highlighted in a study by Ezeigwe [26] that drew attention to the challenges of achieving affordable housing. Therefore, there is the need for new materials development using waste that is readily available, ecofriendly and can be used as an alternative to traditional aggregate to produce low-cost sandcrete blocks as well as help to achieve responsible consumption [27]. Researchers are currently examining the prospects of incorporating agro-based waste materials as suitable alternatives to natural aggregate in composite materials such as concrete, mortar and sandcrete block production. Agro-based materials are reported to be suitable in the production of composite lightweight concrete [23]. Robert et al. [28] explored the possibilities of partially replacing the fine aggregates in masonry blocks with both treated and untreated coconut husk.

Periwinkle shells are one such marine waste material that can be used as an alternative, sustainable material to replace raw materials to reduce pollution and production costs and to limit the problems of land-filling of generated wastes [5,29]. Periwinkles are small greenish-blue marine snails with spiral conical shells and a round aperture [30]. Periwinkle shells are mostly found in riverine/coastal regions in countries such as Nigeria, and are widely distributed in littoral drifts and sandbanks [31]. In Nigeria, the commonly available species of periwinkles are *Pachmellania* spp. and *Tympanostomus* spp., and they are mostly consumed as food; however, the outside shells are discarded as waste into the environment [30,31,32]. The discarded shells are considered an environmental nuisance because of the resultant unpleasant odor and unsightly appearance in open dumpsites but these shells can be recycled as full or partial replacement for aggregates in concrete and sandcrete blocks [5]. Studies by Soneye et al. [2], Otunyo et al. [33] and Agbede and Manasseh [34] investigated the potential utilisation of periwinkle shells as an alternative material to raw nonrenewable aggregates in production of composite sandcrete blocks and concrete. It was reported by Soneye [2] that periwinkle shells can be used up to 30% as replacement for natural aggregate in producing concrete of good strength. Otunyo et al. [33] also pointed out that crushed periwinkle shells can be deployed as fine aggregate replacement in making of lightweight concrete, especially in areas with the shells in abundance, similar to the reported recommendations from the study of Agbede and Manasseh [34] that the strength of concrete with periwinkle shells is similar to that of conventional concrete.

In a recent study by Ogundipe et al. [23], it was opined that both periwinkle and palm kernel shells can be combined in making lightweight structural concrete. Meanwhile, Dauda et al. [35] considered periwinkle shell residues to have pozzolanic cementing capacity and be beneficial as a stabilizing agent for soil improvement, while Odeyemi et al. [36] suggested the combination of periwinkle shells and sawdust materials in the production of quality particle boards of adequate strength. Yawas et al. [37] in their study reported that particles from crushed periwinkle shells can be effectively deployed in the development of brake pads for automobiles. In [38], it was concluded that the combined use of periwinkle shell and lateritic sand is appropriate for making of load-bearing blocks.

This current research examined the potential of recycling and incorporating the periwinkle shell waste as one of the constituent materials in the production of sandcrete blocks that will be beneficial in terms of achieving a cleaner environment through recycling and raw resources conservation, that might also lead to cost and energy savings by producing low-cost lightweight sandcrete blocks made with cement, sand and crushed periwinkle shells. Sandcrete composite contains no coarse aggregate material in its production, but is a composite material, mostly made up of cement binder and sand with addition of water to form sandcrete composite, that can be moulded into different shapes of different sizes [39]. Sandcrete blocks are produced either as solid or hollow rectangular types. The hollow sandcrete blocks are made up of voids that are extruded from top to the bottom and occupy around one third of the volume of blocks, unlike the solid sandcrete blocks, which do not have any form of void in them. Sandcrete blocks are commonly utilised in the production of load-bearing and non-load-bearing masonry walls [40]. Sandcrete blocks are mostly produced in sizes that can be easily handled on site. The most commonly available sizes are 450 mm × 225 mm × 225 mm, and 450 mm × 150 mm × 225 mm for external and internal use, respectively [17]. As Pitroda et al. [41] note, in building construction, there are two main types of walls; non-load-bearing and load-bearing walls. Examples of non-load-bearing walls that will not support any loads from the building, besides their own weight, include partitioning walls used for internal spaces within a building that carry no load and are mostly internal walls. Meanwhile, the load-bearing walls can also perform the same function; that is, they are walls used for partitioning of internal spaces within a building, but that are also used to support load and are often times the outer walls used to transmit the weight of the roof and the upper floors to the foundation [41].

From some of the earlier mentioned studies, periwinkle shell wastes are often used in both concrete and sandcrete composite mixes without crushing the shells. However, it is necessary to examine the possibility of deploying periwinkle shell by crushing it into fine particle sizes. This will help to further expand knowledge in the possible recycling and utilisation of crushed periwinkle shell wastes in building materials. Consequently, this current study investigated the suitability of recycling crushed waste periwinkle shells as a viable alternative to river sand and stone dust in the production of ecofriendly hollow sandcrete blocks that could be used in building construction as part of a strategy to achieve responsible consumption of raw resources. The periwinkle shells were crushed to fine particle sizes that were similar to sand and satisfactory for sandcrete blocks. The specific objectives of the study were to establish the physical and chemical properties of the crushed periwinkle shells, and determine the effect of incorporating the crushed periwinkle shells on the weight, density, strength performance and water absorption tendency of ecofriendly hollow sandcrete blocks.

### Research Significance

Due to the increasing demand, exploitation and consumption of nonrenewable natural aggregates such as river sand for construction, this current study examined the suitability of recycling waste periwinkle shells as a viable alternative to fine aggregates in the production of sandcrete blocks as part of the sustainable development goals in achieving natural resources conservation through responsible consumption. Therefore, this current paper aims to further expand understanding in this area by examining the effect of incorporating the crushed periwinkle shells as a partial substitute for river sand and stone dust on the weight, density, strength performance and water absorption tendency of hollow sandcrete blocks.

## 2. Materials and Methods

### 2.1. Materials

**Figure 1:**
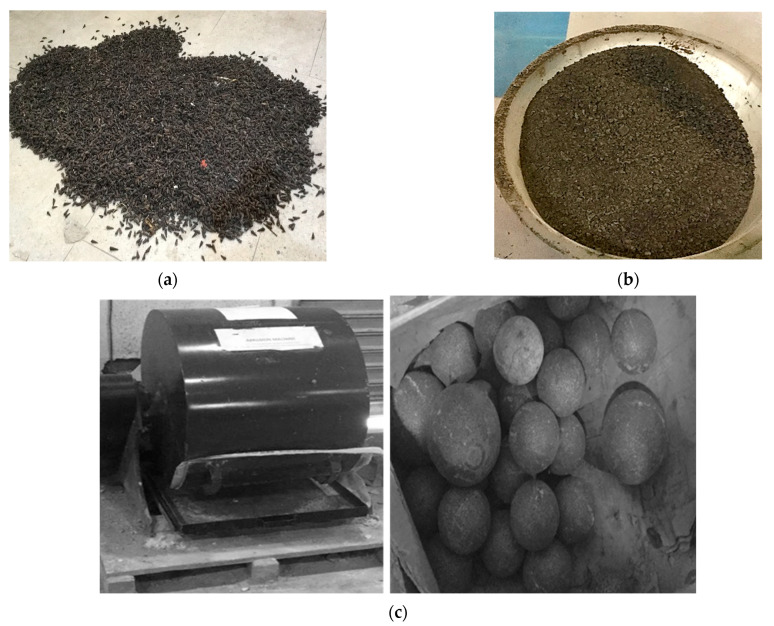
(**a**) Periwinkle shells; (**b**) crushed periwinkle shells; (**c**) laboratory ball milling crusher with steel balls.

The materials used for this research included Portland cement of grade 42.5 bought from a local retail store within Ota town, Ogun State. The river sand passed through a sieve size of 4.75 mm and was obtained from Abeokuta, Ogun State. Stone dust, otherwise called quarry dust, was obtained from a quarry site in Abeokuta in Ogun state. Meanwhile, the periwinkle shells (Figure 1a) were obtained locally from Badagry, Lagos State. Before crushing, the periwinkle shells were first soaked in warm water at about 40 °C for about 2 days to remove dirt, soil and any organic materials present in the shells. These periwinkle shells were then further treated by soaking for 24 h in a 2% solution of sodium hydroxide (NaOH). The soaking in NaOH solution was intended to improve absorbent capacity of the shell after crushing and also remove any unwanted particles that could not be removed by water soaking. The shells were then allowed to sufficiently dry at room temperature conditions before milling. The periwinkle shells were then crushed to a minimum size of 2.45 mm, as presented in Figure 1b, using a laboratory ball milling crusher with steel ball (Figure 1c). The crushed shells were carefully bagged to protect against external moisture and debris. Portable water used for mixing was obtained from the materials laboratory of the department of civil engineering, Covenant University.

Physical properties including the specific gravity, bulk density, water absorption tendency and moisture content of the aggregate materials and crushed periwinkle shells (CPWS) were determined according to BS standards. These tests are important; for instance, the specific gravity test gives the ratio of the mass of the aggregates to a standard or proposed substance of the same given volume with the aim to discover the suitability of the aggregates used in the sandcrete block production process [40]. The water absorption shows the extent to which an aggregate may be susceptible to water. The particle size distribution of the sand, stone dust and CPWS materials was conducted using the sieve analysis approach to confirm the size range of particles present in the aggregates, and also to obtain the particle size distribution, coefficient of uniformity and curvature. The outcomes of the particle size distribution tests on the materials are presented. Non-destructive X-ray fluorescence (XRF) was used to determine the chemical compositions of the cement and CPWS materials used in this study.

### 2.2. Method—Mix Design and Batching

In this study, the batch proportioning of the constituent materials for the hollow sandcrete blocks was carried out by weighing three (3) batches in the laboratory. A constituents design mixture of 1:6 (cement: aggregate) was adopted at a constant water–cement (w/c) ratio of 0.35. The mix ratio of 1:6 was adopted as it is similar to what is generally employed by most local sandcrete blocks industry in Ogun state, and to achieve a realistic onsite simulation. The first batch was the control samples comprised of sand and stone dust, without crushed periwinkle shell (CPWS). The second batch involved the use of the three constituents: sand, stone dust and crushed periwinkle shell (CPWS). River sand was partially replaced by the CPWS using percentage replacement levels of 5, 10, 15 and 20% with a constant amount of cement, w/c ratio and stone dust. Meanwhile, the third batch also used all the three constituent materials; that is river sand, stone dust and crushed periwinkle shell (CPWS). In the third batch, the CPWS was used to partially replace stone dust in the sandcrete mixes using the same percentage replacement levels of 5, 10, 15 and 20% while the river sand, cement and w/c ratio remained constant. The minimum design strength expected to be achieved was 2.5 N/mm^2^. Table 1 depicts the adopted mix proportion in the production of the hollow sandcrete block samples.

### 2.3. Mixing, Sample Preparation and Curing

**Figure 2:**
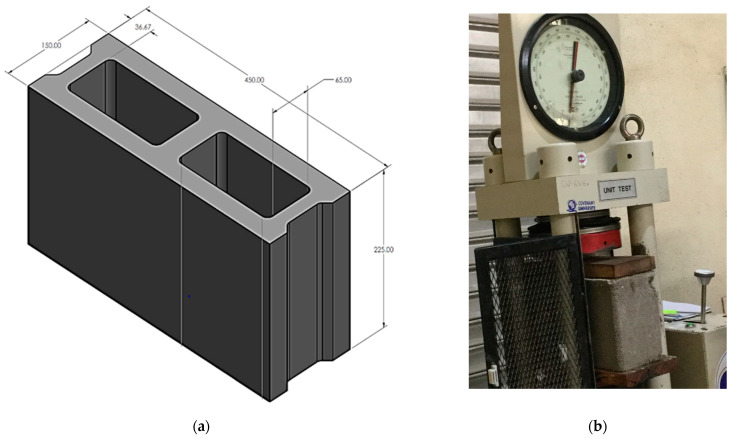
(**a**) Hollow sandcrete block; (**b**) compression test on block sample.

The mixing of the constituent materials and compaction of the sandcrete blocks was carried out manually. The sand, stone dust and CPWS materials were first measured and then evenly mixed to obtain uniform mixture before addition of cement and water. Steel moulds of 450 × 225 × 150 mm^3^ (Figure 2a) dimensions were used for producing the hollow sandcrete blocks, and the moulds were oiled for easy removal of the samples.

A total of 72 sandcrete block samples was produced and each of the block samples was placed on a wooden plank, covered with a damp sack and then laid in a place free from deleterious materials and excess moisture. The covering was done to prevent excessive moisture loss from the samples, and the sacks were removed after 24 h. The curing process was by wetting the sandcrete blocks samples every morning and evening with water up to the testing day for a period of 7 and 28 days at room temperature. The samples were properly labelled to ease identification.

#### Samples Testing

The tests reported include the weight, bulk density, compressive strength and water absorption capacity. They were carried out on the hardened hollow sandcrete block samples to determine their suitability for non-load-bearing and load-bearing applications based on the national specifications NIS 87 [42] standard. The NIS 87 [42] and BS 5628 [43] recommend a compressive strength of 2.5 N/mm^2^ to 3.45 N/mm^2^ for non-load-bearing and load-bearing sandcrete blocks, respectively [44]. These tests on various mix ratios were conducted at the Structures and Materials laboratory of the department of civil engineering, Covenant University, Nigeria. The mean value of three samples was measured for each hollow block property for each of the tested parameters. Weighing balance was used to determine the weight and bulk density of the hollow sandcrete block samples after 28 days of curing. The compressive strength test, which is the ability of the block to resist applied load without failure, was done in compliance with the BS standard. It is one of the various parameters for determining the quality of sandcrete blocks for construction purposes. The compression tests were conducted by placing a smooth wooden surface as base plate at the base of the sandcrete block samples, and also at the top of the block samples to ensure even distribution of the applied load during testing (Figure 2b). The tests on the samples were carried out to failure and the maximum applied load was measured, while the compressive strength of the blocks was computed by dividing the obtained maximum applied load at failure by the cross-sectional area of the hollow sandcrete blocks, as seen in Equation (1).

(1) fc=PA

where *P* = applied maximum load (N) and *A* = effective area (mm).

Moreover, the water absorption tests on the hollow sandcrete blocks were done to determine the water intake tendency of the blocks, which will indicate the durability of hollow sandcrete blocks, especially those produced with CPWS. The water absorption tendency of the samples was determined using the mean of triplicate hollow sandcrete block samples. The samples, after being placed in water for 28 days, were removed and weighed, then oven-dried to a constant mass at a constant temperature of 115 °C. The differences in weight were used to estimate the water absorption rates from Equation (2)

(2) $$water\;absorption\;rate\;\left(\%\right)=\frac{w_{2}-w_{1}}{w_{1}}\times 100$$

where *w*_1_ is the mass of the dry samples and *w*_2_ is the mass of wet samples.

## 3. Results and Discussion

### 3.1. Materials—Chemical Composition

The chemical oxide composition of the cement and crushed periwinkle shells (CPWS) is presented in Table 2. The results showed that the concentration levels of major oxide compounds such as SiO_2_, Fe_2_O_3_, Al_2_O_3_, MgO and CaO in the CPWS materials were somewhat similar to those of cement, as depicted. However, while the findings indicated that both the cement and CPWS contained relatively high concentration of CaO, the results did not show any possible presence of toxic compounds that could leach to the environment. However, the silica content in the CPWS was higher compared to the cement. This is similar to the findings of Ohimain et al. [45] and Ogundipe et al. [23]; thus suggesting that adopting crushed periwinkle shells in cement-based composites will not lead to leaching of toxic organic compounds into the environment.

### 3.2. Materials—Physical Properties of Aggregates

**Figure 3:**
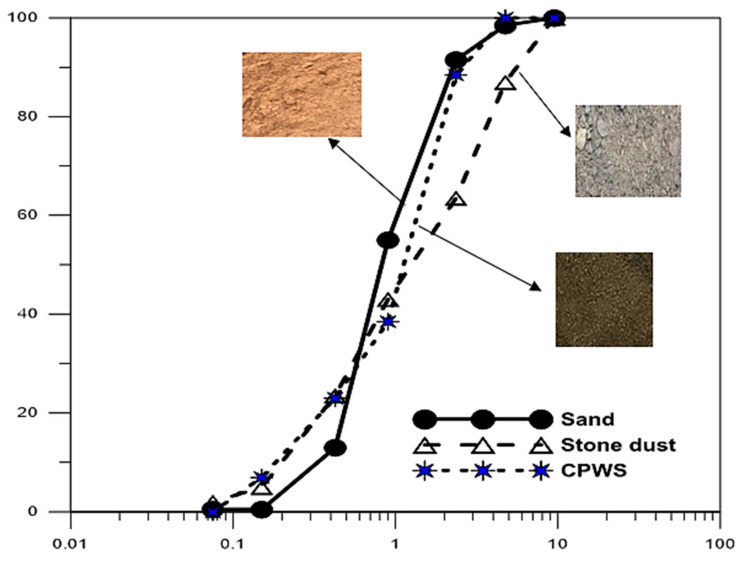
Particle size distribution for sand, stone dust and CPWS.

The results of testing of the physical properties of the aggregates are important to understand how individual aggregate properties influence the overall performance of the produced hollow sandcrete block samples. Figure 3 shows the particle size distribution (PSD) for the river sand, stone dust and the CPWS materials, while Table 3 contains the uniformity coefficients obtained for each of the aggregates used in the production of the hollow sandcrete blocks, as deduced from the PSD plot for each material. The plots show that the aggregates used for producing the hollow sandcrete block samples were suitably well graded. The coefficient of uniformity (Cu) for the river sand gave a value of 9.58 which, being greater than 6, indicated that it was well graded and within the range suggested by Odeyemi et al. [46] and Ogundipe et al. [23] for a fine aggregate material used for producing hollow sandcrete blocks and concrete, respectively. Moreover, the coefficient of curvature (Cc) for the river sand material fell within the value ranges of 1–3, hence indicating that the river sand material was suitable for making hollow sandcrete blocks. Moreover, the coefficients of uniformity (Cu) for the crushed periwinkle shells (CPWS) and stone dust materials were >4, with values of 6.58 and 8.75, respectively. The coefficient of curvature (Cc) values for the CPWS and stone dust materials also fell within the range of 1–3. Therefore, these results indicate the material particle sizes to be well graded and suitable for the production of the hollow sandcrete blocks used in this study.

Table 4 depicts the obtained results on the physical properties and maximum size of the aggregate materials, which included the river sand, stone dust and CPWS used for producing hollow sandcrete block samples. As shown, the obtained values for specific gravity for the sand and stone dust materials were within the acceptable ranges. The specific gravity values of aggregates were expected to fall within the range of 2.3–2.9 [47]. Meanwhile, the recorded specific gravity value of 2.13 for the CPWS material was observed to be lower compared to the sand and stone dust values of 2.39 and 2.58, respectively, thus not within the recommended specific gravity range. The specific gravity results shows that CPWS aggregate may be considered lighter compared to sand and stone dust aggregate. This was also revealed in the bulk density values recorded for the sand, stone dust and CPWS aggregate materials used in the production of the hollow sandcrete block samples. The CPWS recorded the lowest value of 1.566 kg/m^3^, followed by sand with a value of 1.588 kg/m^3^ and stone dust with a value of 1.613 kg/m^3^. The results are closely similar to the reported findings of Ameh et al. [48]. The results on water absorption of the materials showed that CPWS recorded the highest water intake tendency of 8.52%, followed by the stone dust materials (3.54%) and sand (1.13%). The results suggest that an increase in the percentage replacement levels of sand or stone dust with CPWS may possibly increase the water intake tendency of the hollow sandcrete block samples, with possible influence on the strength performance of the hollow sandcrete blocks. The moisture content values of the sand, stone dust and CPWS were all within the 10% value recommended by ACI [47], although the CPWS particles recorded the highest moisture content value, as presented in the table, compared to sand and stone dust materials. The values indicted that the aggregates including the CPWS material utilised are suitable for producing hollow sandcrete blocks for construction purposes.

### 3.3. Hollow Sandcrete Block—Weight and Density

**Figure 4:**
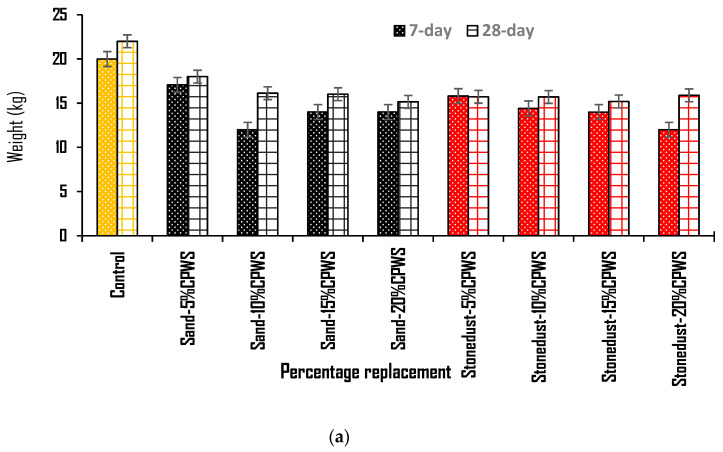

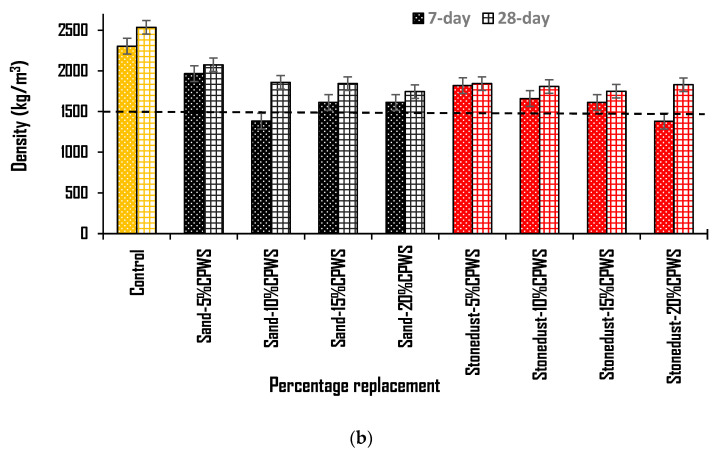
(**a**) Weight of hollow block samples at 7 and 28 days. (**b**) Density of hollow block samples at 7 and 28 days.

Figure 4a shows the results of the weight tests of the produced hollow sandcrete blocks samples. The plot depicts a reduction trend in the weight with increased replacement of sand and stone dust. However, for the hollow sandcrete block samples produced with replacement of stone dust with CPWS content, the weight was noted to be somewhat constant for all replacement levels of 5–20% after 28 days. A similar constant weight was recorded for hollow sandcrete block samples produced with replacement of river sand with approximately 10–20% CPWS content after 28 days. The plot shows a general decrease in the sandcrete weight with increasing percentage content of CPWS as a partial substitute for sand and stone dust. The control recorded the highest weight of 22.0 kg followed by the sample containing 5% CPWS as sand replacement (sand 5% CPWS) with weight of 18.0 kg. The observed weight reduction can be attributed to the low specific gravity value of the CPWS material compared to that of river sand and stone dust. This indicates that CPWS can be beneficially deploy as material to achieve lightweight hollow sandcrete blocks for lightweight construction and is similar to the study results of Sojobi et al. [8]. Meanwhile, Figure 4b shows the results of the estimated density tests of the hollow sandcrete block samples. The recommended minimum value expected for blocks made with lightweight aggregate is 1500 kg/m^3^, as stated by NIS 87 [42]. The plot display concerning the sandcrete blocks shows that almost all the samples achieved the density value as stipulated in NIS 87 [42] at 28 days’ curing age, but two of the block samples produced were below the 1500 kg/m^3^ value at 7 days curing age. A similar decrease in the density of the sandcrete blocks was observed as the CPWS content increased compared to the control. The control recorded the highest weight and density of 22.0 kg and 2534.56 kg/m^3^, respectively. However, the weight and density of other block samples containing CPWS as replacement for sand and stone dust were noted to be somewhat constant, as seen from the plot, except at 5% sand replacement with CPWS, which recorded 18.0 kg and 2073.73 kg/m^3^ for weight and density, respectively. From the recorded weight of sandcrete blocks and their various bulk densities, the results showed that the greater weight exhibited higher bulk density and the low-weight samples exhibited low bulk densities. This is consistent with the findings of Ogundipe et al. [23] and Odeyemi et al. [36] that periwinkle shells resulted in density reduction when incorporated in composite material, and is also somewhat similar to the reported findings of Dadzie and Yankah [49] and Sojobi et al. [8] on achieving lightweight construction. Hence, the lower the weight of the blocks, the lower the bulk density of the sandcrete blocks.

### 3.4. Hollow Sandcrete Block—Compressive Strength

**Figure 5:**
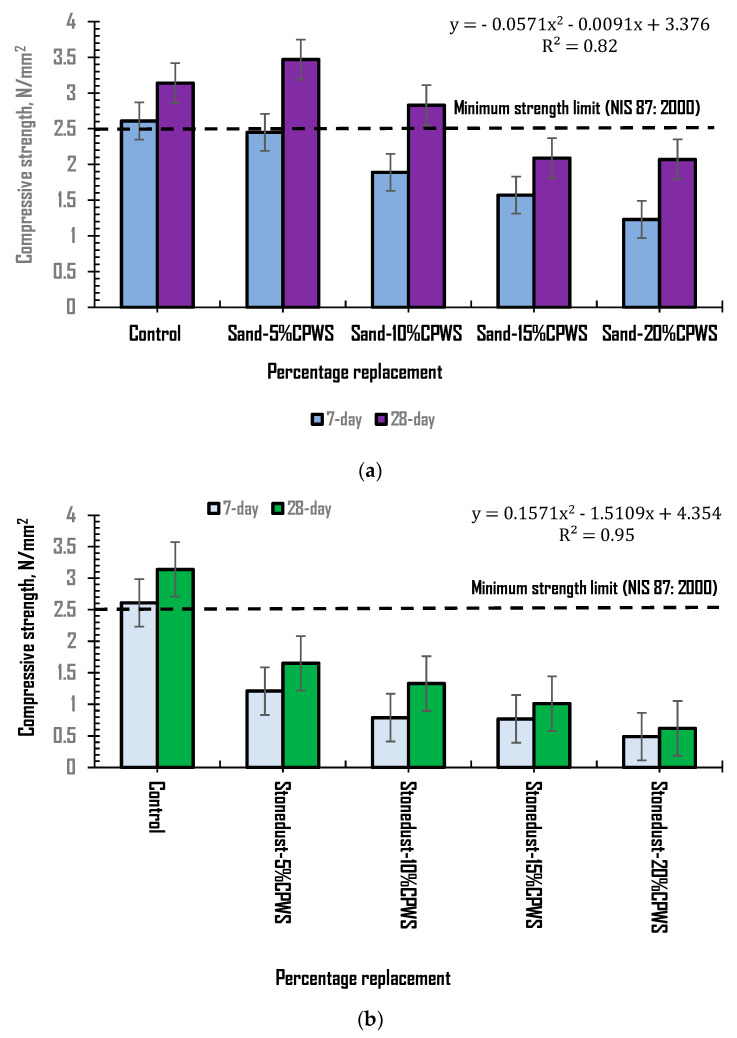
(**a**) Strength development of samples with CPWS as sand replacement. (**b**) Strength development of samples with CPWS as stone dust replacement.

Figure 5a presents the characteristic strength development of the control and hollow sandcrete blocks produced with CPWS as partial replacement for sand at percentage levels of 5, 10, 15 and 20%, with other constituent materials constant, at 7 and 28 days’ curing. The plot shows a reduction in the compressive strength of the samples as the CPWS content increased compared to the control samples. Still, the block samples with 5% and 10% sand replacement CPWS showed good compressive strength values of 3.47 N/mm^2^ and 2.83 N/mm^2^, respectively, compared to the control value of 3.14 N/mm^2^ at 28 days’ curing age. The recorded values were within the stipulated values of 2.5 N/mm^2^ and 3.45 N/mm^2^ recommended by the Nigerian Industrial Standard [42] on hollow sandcrete blocks for both load-bearing and non-load bearing purposes. This suggests the possibility of using the blocks made with crushed periwinkle shells at low content for load-bearing wall unit construction. Meanwhile, the plot block samples containing 15 and 20% CPWS content as sand replacement recorded compressive strength values of 2.09 N/mm^2^ and 2.07 N/mm^2^, respectively. These values were below the recommended values, thus suggesting that these block samples can only be deployed for non–load bearing purposes. The hollow sandcrete block samples made with 5% CPWS as replacement for sand attained the highest compressive strength value compared to the control with an increase of 10% in compressive strength.

Further, Figure 5b also showed the compressive strength results for control and hollow sandcrete blocks produced with CPWS as partial replacement for stone dust at similar percentage levels of 5, 10, 15 and 20% with other constituent materials kept constant at 7 and 28 days’ curing. The plot shows a significant decrease in the compressive strength of the block samples as the CPWS content increased compared to the control. The recorded compressive values at 5, 10, 15 and 20% replacement of stone dust with CPWS content were 1.65 N/mm^2^, 1.33 N/mm^2^, 1.01 N/mm^2^ and 0.62 N/mm^2^, respectively compared to the control (3.14 N/mm^2^) at 28 days’ curing age. These values fell below the minimum recommended compressive strength values of 2.5 N/mm^2^ [42]. The decrease in the compressive strength relative to the control was more than a 45% reduction, indicating the prominent influence of the stone dust in making quality hollow sandcrete blocks. It can be inferred from Figure 5a,b that partially replacing the stone dust content with CPWS in hollow sandcrete blocks will negatively affect strength compared to partially replacing the sand content with CPWS. This can be attributed to the filling ability effect the stone dust had in the hollow sandcrete block samples, which could not be sufficiently replaced by the CPWS material. The plot shows that hollow sandcrete blocks for load bearing applications can be produced by limiting the CPWS content to less than 10% as sand replacement, while keeping the stone dust constant.

### 3.5. Hollow Sandcrete Block—Water Absorption

**Figure 6:**
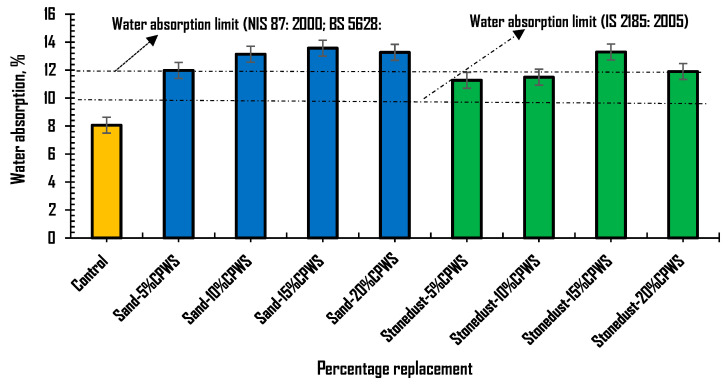
Water absorption rate of sandcrete block samples with CPWS as sand and stone dust replacement.

Figure 6 depicts the results of the water absorption tendency tests of the hollow sandcrete block samples containing CPWS as replacement for sand and stone dust relative to the reference hollow sandcrete block. The water absorption of sandcrete block samples was estimated after 28 days of curing age when the blocks were expected to have fully attained the desired strength. The plot shows the results obtained, which were also related to the recommended limits of 12% stated in NIS 87 [42] and BS 5628 [43], and 10% stated in IS 2185 [50]. These standards recommend the maximum water intake limit allowed for hollow sandcrete blocks for masonry application. The plot shows that the reference sandcrete blocks, that is, blocks produced without CPWS, recorded a lower rate of water absorption compared to hollow sandcrete block samples containing CPWS, thus meeting the NIS 87 [42], BS 5628 [43] and IS 2185 [50] limits of 12% and 10%, respectively. Moreover, the plot also indicates a gradual increase in the water absorption rate of the sandcrete blocks as the percentage amount of CPWS content increased for both sand and stone dust replacement. It can therefore be inferred that an increase in the percentage replacement of sand or stone dust with crushed periwinkle shells material will likely lead to an increase in the water absorption tendency of the sandcrete block samples. The plot shows that the obtained water absorption values surpassed the limit of 10% set by IS 2185 [50], while the sandcrete blocks containing high CPWS content surpassed the 12% set by NIS 87 [42]. Blocks containing CPWS at 10%, 15% and 20% replacement of sand and 15% replacement of stone dust recorded values of 13.14%, 13.57%, 13.27% and 13.30%, respectively, above the recommended values, or an increase of about 68% in the water absorption rate relative to the control. The observed water intake rate for block samples containing a low CPWS content of 5% as replacement for sand and stone dust was within the 12% recommended by NIS 87 [42] and BS 5628 [43]. The increase was about 40% at low CPWS content. The obtained results are somewhat similar to those of studies reported by Odeyemi et al. [51] and Omopariola [52] on the tendency of sandcrete blocks to absorb and retain water. Moreover, findings on the physical properties of the CPWS material itself showed high values of moisture content and water absorption tendency, which could have further contributed to the increasing water intake rate as the CPWS content increased in the sandcrete block samples. Although this study did not consider particle board, the results are not consistent with findings of Odeyemi et al. [36] on water absorption reduction in particle board produced with periwinkle shell residue and sawdust. Therefore, good quality hollow sandcrete blocks incorporating CPWS and having a low water absorption rate within acceptable range can be produced by limiting the CPWS content to not more than 10% replacement level.

## 4. Conclusions

This study investigated the utilisation of crushed periwinkle shell material as a partial substitute for fine aggregates (stone dust and sand) in production of ecofriendly hollow sandcrete blocks for masonry works to achieve sustainable construction. The study focused on the physical properties of the CPWS material and their effects on the properties of hollow sandcrete blocks. The following conclusions can be drawn from this study:

1. The material characterisation of the CPWS showed that the material has high values of moisture content and water absorption rate compared to sand and stone dust materials. However, the recorded values for the specific gravity and bulk density were very low compared to those of sand and stone dust materials. This indicated that the CPWS material has capacity to absorb and retain water but will be most suited to achieve a lightweight construction. Meanwhile, the chemical composition of both CPWS and cement are closely similar with relatively high concentration of CaO, and no possible presence of toxic compounds that can leach into the environment.
2. Further, the particle size gradation plot of CPWS indicates the material is well graded, as also seen from the coefficient of curvature (Cc) and coefficient of uniformity (Cu) values, hence showing the utilisation of CPWS is possible in producing homogenous sandcrete block mixes.
3. The weight and hardened density of the produced hollow sandcrete blocks decreased as the percentage of CPWS content increased but remained somewhat constant as the replacement levels increased. However, the obtained average weight and density of the hollow sandcrete blocks at 28 days were within the range for lightweight sandcrete blocks.
4. The results on compressive strength suggested that CPWS will be most suitable to be deployed as a partial substitute for sand while stone dust material is kept constant in the production of sandcrete blocks. The outcomes indicated a significant strength reduction of the block samples for mixes containing CPWS as partial replacement for stone dust, far below the targeted strength. However, mixes containing 5% and 10% CPWS as a partial substitute for sand were able to achieve the targeted strength of 2.5 N/mm^2^ at 28 days. The results imply that block samples containing CPWS as replacement for sand with 100% stone dust tend to produce hollow sandcrete blocks of equal strength to the control compared to samples containing CPWS as replacement for stone dust with constant quantities of sand.
5. The outcome of the water absorption results indicated an increase in the water absorption rate of the hollow sandcrete block samples as the CPWS content increased.

This study further highlights the key role the construction industry can play towards achieving a cleaner and sustainable built environment, as well as responsible consumption of natural resources in line with the sustainable development goals. The study shows the potential of utilising crushed periwinkle shells as an alternative constituent aggregate in the production of sustainable lightweight hollow sandcrete blocks that can be used for construction. The results indicated that CPWS may be considered as an alternative aggregate to sand at 5% optimum replacement level and 100% stone dust to produce lightweight hollow sandcrete block suitable for both load and non–load–bearing masonry works. Additionally, increasing the CPWS content to 10% optimum replacement level can be possibly considered but should be limited to lightweight hollow sandcrete blocks produced for internal uses and for non-load-bearing masonry works.

### Recommendations for Future Studies

In future studies, it will be necessary to investigate the potential of other locally available agricultural or marine-based waste materials as an alternative to traditional natural aggregate in production of sandcrete composites. In addition, it will be required to examine further the durability performance of such sandcrete products containing agricultural or marine-based waste materials as substitutes for natural aggregate. The possible utilisation of CPWS or any marine-based waste material as a substitute for natural aggregate in high-performance lightweight mortar or concrete composites should be further examined, including determination of the influence of the CPWS material’s shape on its properties.

## Figures and Tables

**Table 1:** Sandcrete block constituents mix proportioning.

S/N	Samples Description	Cement (kg)	Sand (kg)	Stone Dust (kg)	CPWS (kg)	w/c Ratio
1	Control	3.00	8.50	8.50	-	0.35
2	Sand-5%CPWS	3.00	8.03	8.50	0.42	0.35
3	Sand-10%CPWS	3.00	7.61	8.50	0.85	0.35
4	Sand-15%CPWS	3.00	7.18	8.50	1.27	0.35
5	Sand-20%CPWS	3.00	6.76	8.50	1.69	0.35
6	Stonedust-5%CPWS	3.00	8.50	8.03	0.42	0.35
7	Stonedust-10%CPWS	3.00	8.50	7.61	0.85	0.35
8	Stonedust-15%CPWS	3.00	8.50	7.18	1.27	0.35
9	Stonedust-20%CPWS	3.00	8.50	6.76	1.69	0.35

**Table 2:** Chemical composition of cement and CPWS.

Composition, %	Materials	
	Cement	CPWS
Aluminum oxide, Al_2_O_3_	4.72	8.30
Silicon oxide, SiO_2_	16.56	31.10
Ferric oxide, Fe_2_O_3_	2.86	4.21
Sulfur trioxide, SO_3_	2.86	0.06
Magnesium oxide, MgO	1.45	0.76
Calcium oxide, CaO	63.48	53.10
Sodium oxide, Na_2_O	0.60	0.02
Potassium oxide, K_2_O	0.10	0.12
Titanium dioxide, TiO_2_	-	0.40
Loss on ignition, LOI	0	2.50

**Table 3:** Particle size distribution of aggregate.

Aggregate	D_10_	D_30_	D_60_	C_u_	C_c_	USCS Classification	
						Group Symbol	Group Name
River sand Stone dust	0.3 0.2	0.6 0.6	2.875 1.75	9.58 8.75	0.42 1.03	SW GW	Well graded Well graded
CPWS	0.19	0.65	1.25	6.58	1.78	GW	Well graded

Cu = D60/D10, while Cc = (D30)^2^/(D10) (D60)

**Table 4:** Physical properties of constituent materials.

Properties	Cement	Stone Dust	River Sand	CPWS
Specific gravity	3.15	2.58	2.39	2.13
Surface area, m^2^/kg	354	-	-	-
Max aggregate size, mm	-	4.00	4.75	2.45
Fineness modulus		-	2.69	
Water absorption (%)	-	3.54	1.13	8.52
Moisture content (%)	-	0.41	0.18	1.30
Bulk density, kg/m^3^	-	1.613	1.588	1.566
